# Author Correction: Overcoming the permeability-selectivity challenge in water purification using two-dimensional cobalt-functionalized vermiculite membrane

**DOI:** 10.1038/s41467-024-45259-2

**Published:** 2024-01-25

**Authors:** Mengtao Tian, Yi Liu, Shaoze Zhang, Can Yu, Kostya (Ken) Ostrikov, Zhenghua Zhang

**Affiliations:** 1 Membrane & Nanotechnology-Enabled Water Treatment Center, Guangdong Provincial Engineering Research Center for Urban Water Recycling and Environmental Safety, Tsinghua Shenzhen International Graduate School, Tsinghua University, Shenzhen, 518055 Guangdong China; 2 School of Environment, Tsinghua University, Beijing, 100084 China; 3 National Engineering Laboratory for Vacuum Metallurgy, Kunming University of Science and Technology, Kunming, 650093 Yunnan China; 4 Institute of High Energy Physics, Chinese Academy of Sciences (CAS), Beijing, 100049 China; 5 School of Chemistry and Physics, QUT Centre for Materials Science, Queensland University of Technology (QUT), Brisbane, Queensland 4000 Australia

**Keywords:** Environmental, health and safety issues, Two-dimensional materials, Heterogeneous catalysis

Correction to: *Nature Communications* 10.1038/s41467-024-44699-0, published online 09 January 2024

The original version of this Article contained an error in Fig. 4. In the original version, the magnetic field values of the x-axis in panel b were incorrectly displayed without a final ‘0’. This has been corrected in both the PDF and HTML versions of the Article.

