# Supplementary material for: Overcoming the permeability-selectivity challenge in water purification using two-dimensional cobalt-functionalized vermiculite membrane
Source: Nat Commun. 2024 Jan 9;15:391. doi: 10.1038/s41467-024-44699-0 (PMC10776859; doi:10.1038/s41467-024-44699-0)
Supplement: Supplementary file 1 — Supplementary Information [file 41467_2024_44699_MOESM1_ESM.pdf]

# Supporting information

## Overcoming the permeability-selectivity challenge in water purification using two-dimensional cobalt-functionalized vermiculite membrane

Mengtao Tian[a], Yi Liu[a,b], Shaoze Zhang[c], Can Yu[d], Kostya (Ken) Ostrikov[e], and Zhenghua Zhang[a,b,e*]

[a]Membrane & Nanotechnology-Enabled Water Treatment Center, Guangdong Provincial Engineering Research Center for Urban Water Recycling and Environmental Safety, Tsinghua Shenzhen International Graduate School, Tsinghua University, Shenzhen 518055, Guangdong, China

[b]School of Environment, Tsinghua University, Beijing 100084, China

[c]National Engineering Laboratory for Vacuum Metallurgy, Kunming University of Science and Technology, Kunming 650093, Yunnan, China.

[d]Institute of High Energy Physics, Chinese Academy of Sciences (CAS), Beijing 100049, China

[e]School of Chemistry and Physics, QUT Centre for Materials Science, Queensland University of Technology (QUT), Brisbane, Queensland 4000, Australia.

*Corresponding author: zhenghua.zhang@sz.tsinghua.edu.cn (Z. Zhang)

# 1. Supplementary methods

## 1.1 Materials and reagents

Vermiculite (VMT, sizes: 1.4–4 mm) was purchased from Xinjiang Yuli Xinlong Vermiculite Co., Ltd., China). Polyvinylidene difluoride (PVDF) (pore size = 0.22 μm) disk membrane was purchased from APNEL Laboratory Technology, Shanghai, China. Oxone ($2KHSO_5 \cdot KHSO_4 \cdot K_2SO_4$) mainly containing active component peroxymonosulfate (PMS, $KHSO_5$) was supplied by Aladdin, China. Cobalt (II) acetate ($Co(Ac)_2$), sodium hydroxide (NaOH), sodium chloride (NaCl), Lithium Chloride (LiCl), Hydrogen Peroxide ($H_2O_2$), tert-butyl alcohol (TBA), p-benzoquinone (p-BQ), 5,5-Dimethyl-pyrroline-N-oxide (DMPO), 2,2,6,6-tetramethyl-4-piperidinol (TEMP), methyl orange (MO), methylene blue (MB), rhodamine B (RhB), phenol, bisphenol A (BPA), carbamazepine (CAR), and oxytetracycline (OXY) were obtained from Macklin, China. Hydrochloric acid (HCl, 36%wt) was purchased from Guangzhou Chemical Reagent Factory, China, while ethanol (EtOH, >99.7%) was bought from Aladdin, China. Ranitidine was obtained from Tokyo Huacheng Industry. Unless otherwise specified, all the chemicals were of analytical grade (purity >98%) and used without any further purification.

## 1.2 Characterization of Co@VMT nanocomposites and Co@VMT membranes

VMT nanosheet, Co@VMT nanocomposites and Co@VMT membranes were characterized. Morphology and thickness of Co@VMT nanocomposites were obtained using atomic force microscopy (AFM, Bruker Dimension Icon, Germany),

and transmission electron microscopy (TEM, JEOL 2100F, Japan). X-ray absorption fine structure (XAFS) spectroscopy was carried out using the RapidXAFS 1M (Anhui Absorption Spectroscopy Analysis Instrument Co., Ltd.) by transmission mode at 20 kV and 40 mA, and the Si (533) spherically bent crystal analyzer with a radius of curvature of 500 mm was used. X-ray diffraction (XRD) ($\lambda$=0.15418 nm, Bruker D8 Advance, Germany) was used to analyze the phase structure of VMT nanosheet, Co@VMT nanocomposites and d-spacing of membranes. The morphology and cross-section images as well as elemental mapping of cross-section of membranes were obtained using a scanning electron microscope (SEM) equipped with an energy dispersive spectroscope (SEM-EDS, Hitachi SU8010, Japan). The atomic composition and chemical structures of Co@VMT membranes were confirmed by X-ray photoelectron spectroscopic (XPS) (PHI5000 Versa Probe-II, Japan) with a monochromated Al K$\alpha$ radiation at 1486.6 eV. The VMT membrane and Co@VMT membrane were analysed using Brunauer-Emmitt-Teller (BET), Barrett-Joyner-Halenda (BJH) methods (Belsorp, Microtrac Bel, Japan), and Horvath-Kawazoe (HK) methods (Belsorp, Microtrac Bel, Japan) for measuring the specific surface area and pore size distribution, respectively. The BET analysis was performed with Ar adsorption/desorption isotherms at 87 K. Total organic carbon (TOC) was measured using TOC analyzer (Shimadzu, Japan), while cobalt concentration was quantified using an inductively coupled plasma mass spectrometry (ICP-MS, Agilent). Electron paramagnetic resonance (EPR) spectra were obtained using MS-5000 spectrometer (Bruker, Germany). Analysis of degradation products

was conducted using liquid chromatography/tandem mass spectrometry (LC-MS, Agilent, USA, 1290UPLC-6550QTOF). The acute toxicity (LC50 or EC50) and chronic toxicity (ChV) of ranitidine and its intermediates to three aquatic taxa, including fish, daphnids, and algae, were predicted using the Ecological Structure-Activity Relationship (ECOSAR) class program (ECOSAR v2.2, EPA).

## 1.3 Experiment measurements

### 1.3.1 Quenching experiments and EPR tests

To determine the role of $SO4^{\bullet-}$, •OH, $^1O_2$, and $\bullet O_2^-$species in the degradation process, quenching experiments were carried out using ethanol (100 mM), TBA (100 mM), TEMP (500 mM), and p-BQ (1 mM), respectively. Additionally, EPR spectroscopy was used to measure the intensity of these ROS (Burker MS-5000, Germany).

### 1.3.2 Determination of PMS decomposition efficiency

Firstly, a test solution containing 100 $g.L^{-1}$ KI and 5 $g.L^{-1}$ $NaHCO_3$ was prepared. Then, 0.2 mL sample and 20 mL test solution were fully mixed and placed for more than 30 min followed by the UV absorbance measurement at 352 nm. The PMS decomposition efficiency, D (%), was calculated using Equation (1):

$$D = \frac{A_0 - A_t}{A_0} \times 100\% \qquad (1)$$

where $A_0$ is the absorbance of the original PMS test solution, and $A_t$ is the absorbance of the sample solution at the specified time point.

## 1.4 Computational methodology

### 1.4.1 DFT

The Vienna Ab initio Simulation Package (VASP) software (version 5.4.1) was applied to carry out the periodic density functional theory (DFT) calculations[1-2]. The ion-electron exchange correlation was described through the Perdew-Burke-Ernzerhof (PBE) exchange correlation functions and the projector-augmented wave (PAW) technique. The long-range dispersion was adjusted using the DFT-D3 with Becke-Johnson damping. The plane-wave expansion's energy cutoff was set at 450 eV. The Monkhorst-Pack method was used to generate the Brillouin zone integration from a Gamma centered 2 × 2 × 1 k-point mesh. All structures were relaxed until the energy changed less than $1 \times 10^{-5}$ eV, with the forces on each atom below 0.01 eV.$Å^{-1}$. The (111) surface of $Co_3O_4$, and (100) and (110) surfaces of $Co(OH)_2$ were constructed and optimized. The adsorption energy ($E_{ads}$) was calculated as:

$$E_{\mathrm{ads}} = E_{\mathrm{system}} - E_{\mathrm{surface}} - E_{\mathrm{PMS}} \qquad (2)$$

where the $E_{system}$ is the energy of the optimized adsorption system; $E_{surface}$ is the energy of the bare surface; $E_{PMS}$ is the energy of an optimized PMS molecule within a 30 × 30 × 30 box.

### 1.4.2 MD

Molecular dynamics (MD) simulations using the Forcite module in Material Studio package were performed to reveal the transport mechanism of PMS, ranitidine, and water within the Co-VMT membrane and VMT membrane[1]. First, 4 PMS, 1 ranitidine and 100 water molecules were added into a solvent module through a

Drieding force field by Amorphous Cell (AC) construction. Then, Building Layers were used to combine the solvent module with VMT and Co-VMT systems which were carried geometry optimization first and then slabbed into (001) configuration. It is worth noting that the transport paths of the solvent module included interlayer and intralayer paths, of which the free spacing was based on  the results of XRD and pore size distribution of VMT and Co@VMT membranes (Fig.1e and Fig.S6), respectively. The combined systems (VMT configuration with the solvent module, and Co-VMT configuration with the solvent module) were carried geometry optimization using Smart Algorithm with the max iteration time of 5000. Then the systems were set at 0. 1 MPa and 300 K to run the whole MD process. After 10 ps NVT and 10 ps NPT performed to prevent the unrealistic structure of the systems, the final 10 ps NVT process was calculated to collect the Mean Square Displacement (MSD) curve to reveal the transport process. During all the MD calculations, the universal force field was selected due to the complicacy of the elements in the systems. We chose a 0.7 fs-time step with 1 frame export every 250 steps (14286 steps in total). The electrostatic and van der Waals summation methods were set as Ewald and Atom based methods, respectively. The cut off distance was set as 12.5 Å with a 0.5 Å buffer width.

## 2. Supplementary Figures

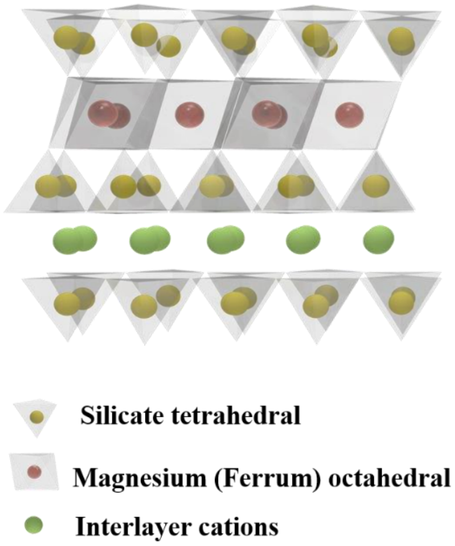


**Figure 1. The macrostructure of bulk VMT.** Schematic of VMT bulk structure stacked by adjacent VMT nanosheets through electrostatic attraction with interlayer cations.

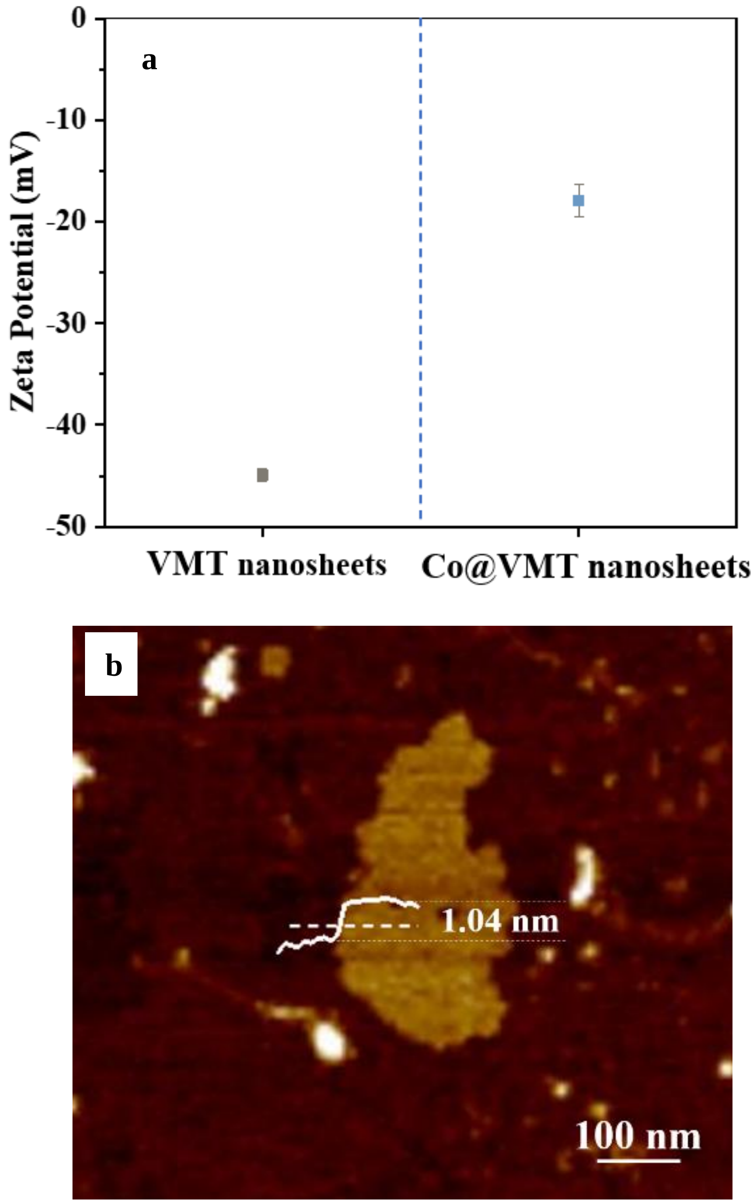


**Figure 2. Zeta potential (a) and AFM (b) characterization.** Zeta potentials of the suspended VMT nanosheets and Co@VMT nanosheets (pH=7, C=0.1 mg.mL$^{-1}$). AFM image with the height profile of Co@VMT nanosheets. Error bars represent standard deviation of three measurements.

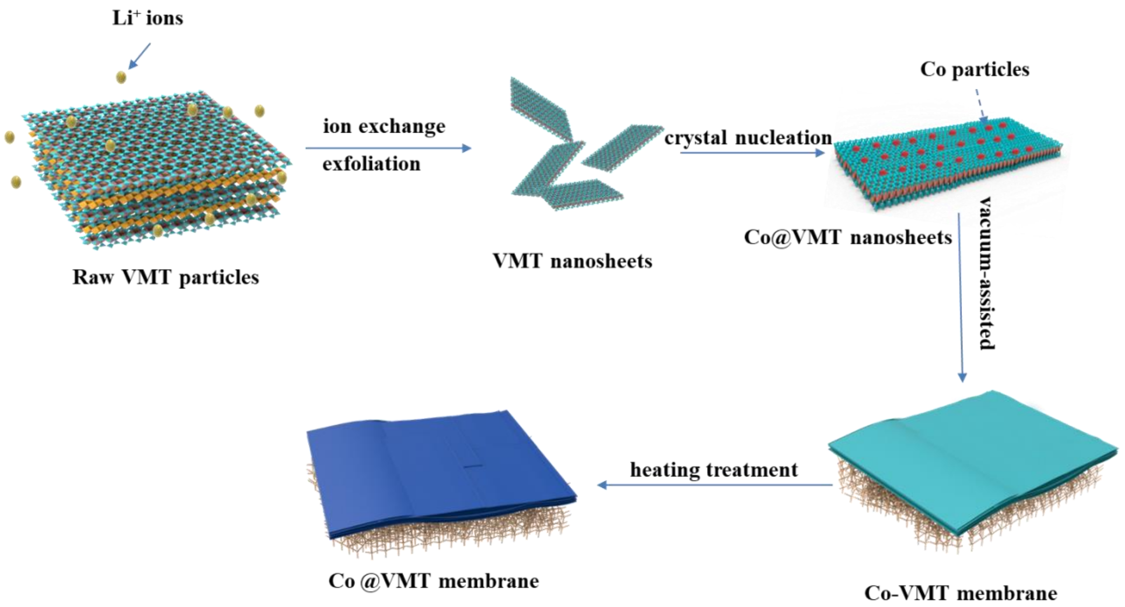


**Figure 3. Fabrication of Co@VMT membrane.** Schematic of the synthetic procedures of Co@VMT nanosheets and Co@VMT membrane.

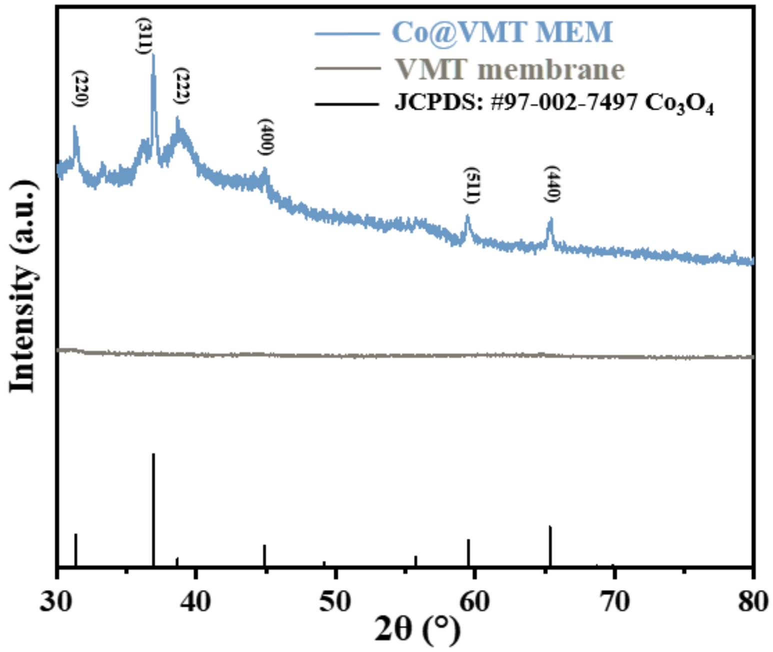


**Figure 4. XRD analysis.** XRD patterns of wetted VMT and Co@VMT membranes at relatively high Bragg angles.

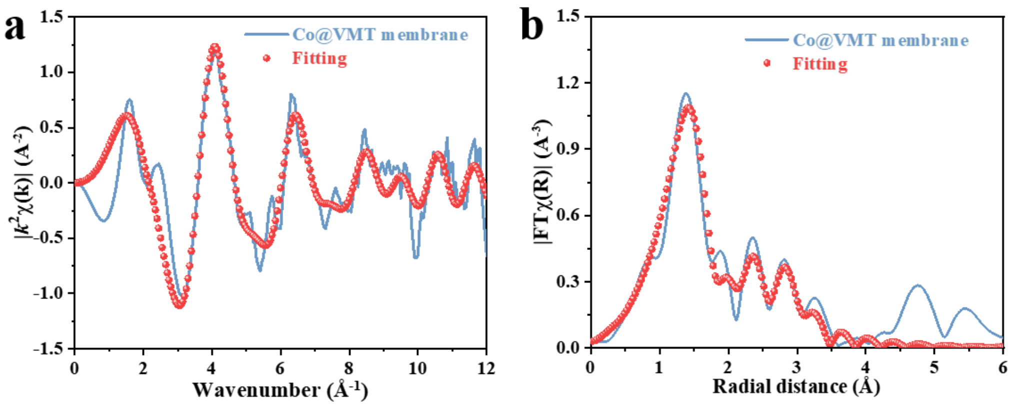


**Figure 5. EXAFS characterization**. Co K-edge EXAFS fitting curves of Co@VMT membrane at k-space (a) and R-space (b).

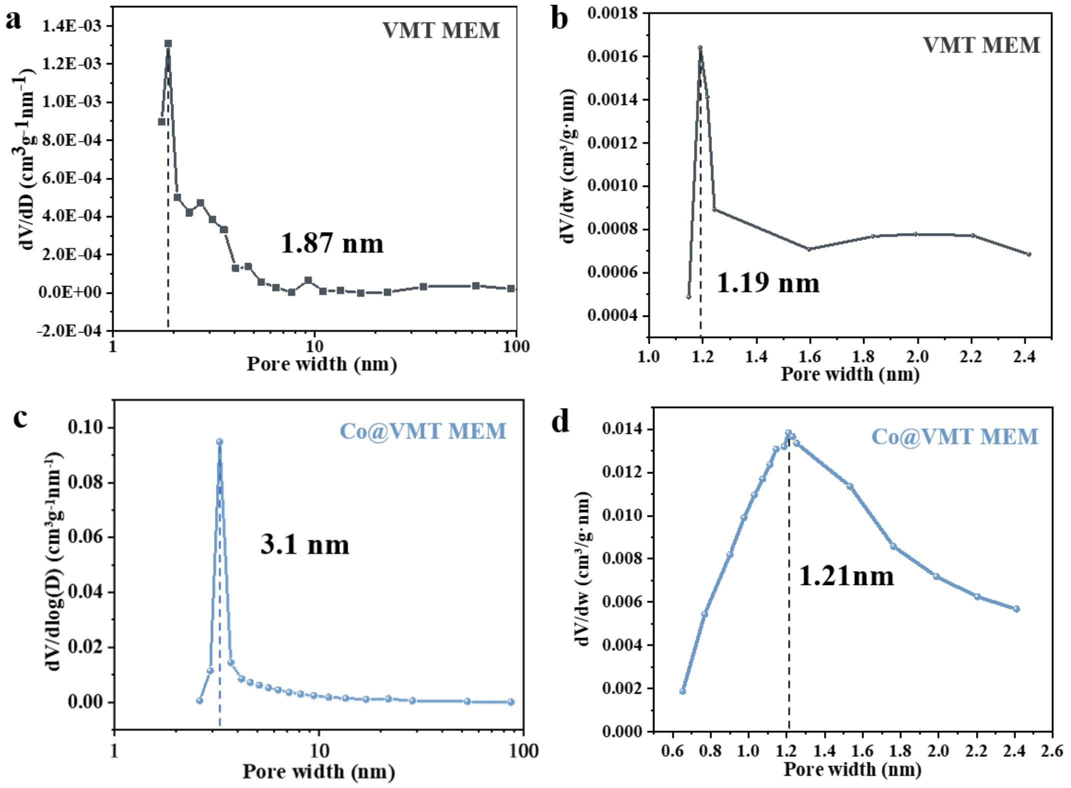


**Figure 6. Pore size distribution analysis**. Barrett-Joyner-Halenda (BJH) and Horvath-Kawazoe (HK) models for pore size distribution analysis for VMT (a, b) and Co@VMT membranes (c, d).

The pore size distribution of VMT and Co@VMT membranes was estimated using the Horvath-Kawazoe (HK) and Barrett, Joyner, and Halenda (BJH) methods. The BJH model is suitable for mesopores and may not work for the estimation of micropores as the Kelvin equation of BJH model is not applicable for pores with size below 2 nm[2]. As such, BJH model is usually applied for the estimation of mesopores with size above 5 nm, while the HK model is commonly used for the quantification of pores (including the micropores) with size less than 5 nm. When we applied the BJH pore size estimation method for the VMT and Co@VMT membranes, it was obvious that pores were mainly located in the range of < 5 nm (Fig. S6a and S6c), which fits the scope of the HK model. Hence, we finally chose the HK model for pore size estimation of VMT and Co@VMT membranes (Fig. S6b and S6d).

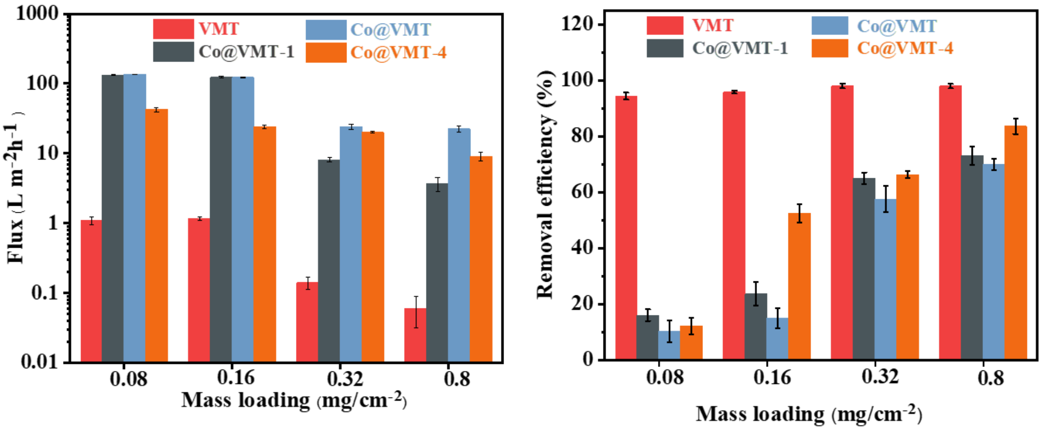


**Figure 7. Membrane permeability-selectivity performance evaluation.** Flux (a) and removal efficiency of ranitidine (b) for VMT and Co@VMT membranes with different mass loading of Co:VMT (10 ppm ranitidine; 1 bar pressure). Error bars represent standard deviation of three measurements.

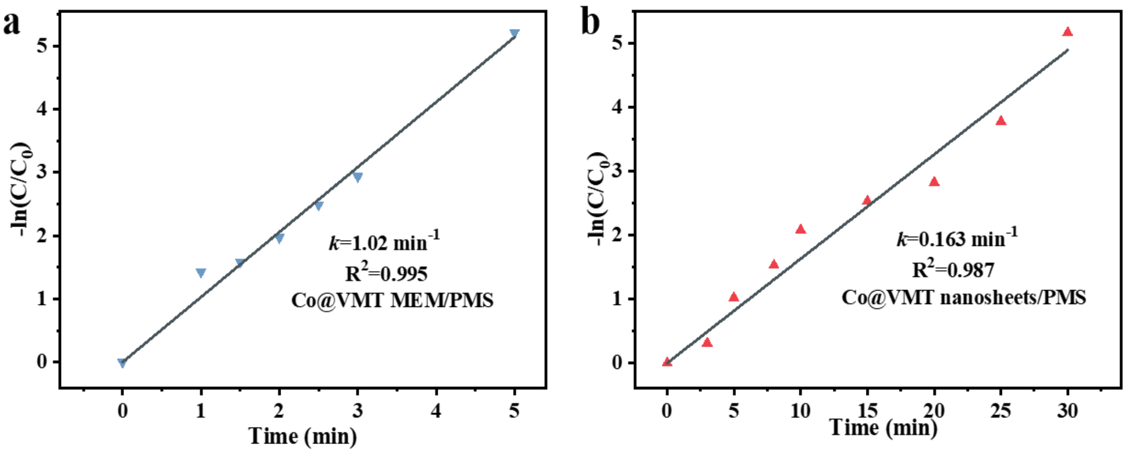


**Figure 8. First-order rate constant analysis.** The fitted first-order rate constant ($k$) of the removal of ranitidine for the Co@VMT MEM/PMS (a) and Co@VMT nanosheets/PMS (b) systems.

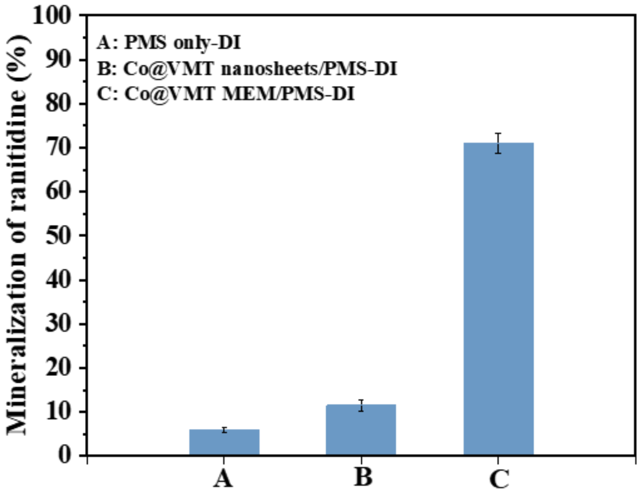


**Figure 9. Mineralization performance of ranitidine.** TOC removal achieved by different systems in 30 min with Milli-Q water as the water matrix. Error bars represent standard deviation of three measurements.

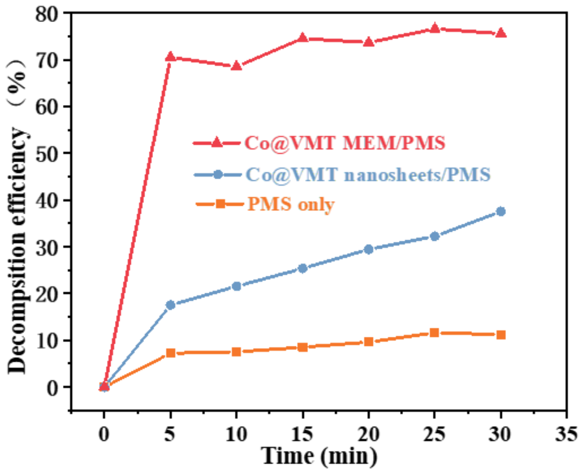


**Figure 10. PMS decomposition performance**. Decomposition efficiency of PMS in different systems. Error bars represent standard deviation of three measurements.

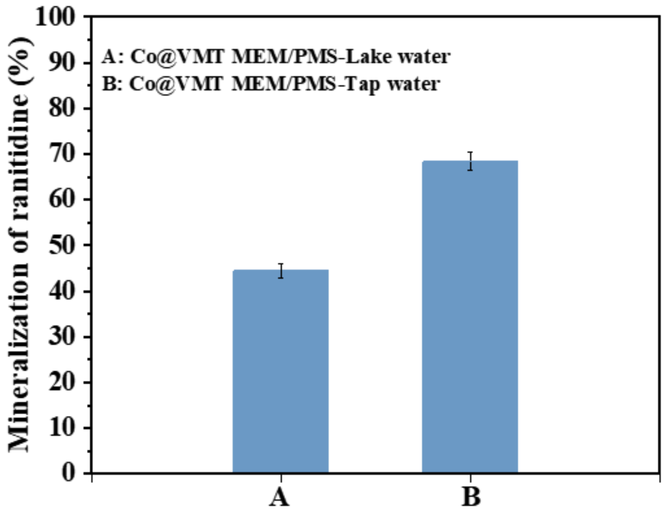


**Figure 11. Mineralization performance of ranitidine.** TOC removal achieved by different systems in 30 min using lake water and tap water as the real water matrices. Error bars represent standard deviation of three measurements.

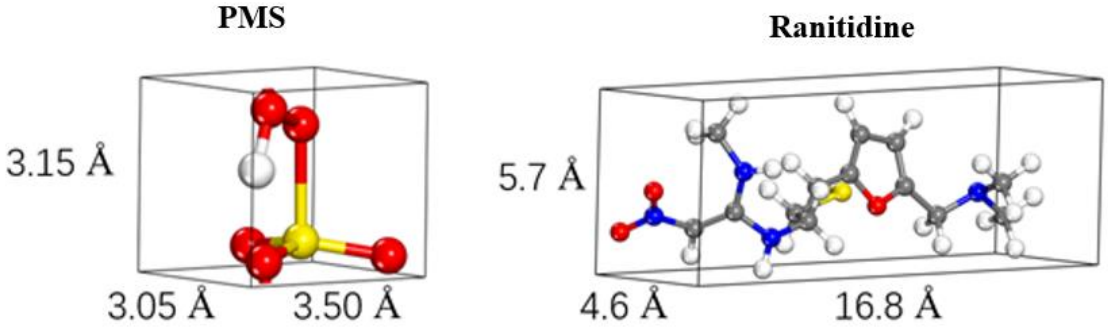


**Figure 12. Molecular size analysis**. The molecular size of PMS and ranitidine molecules.

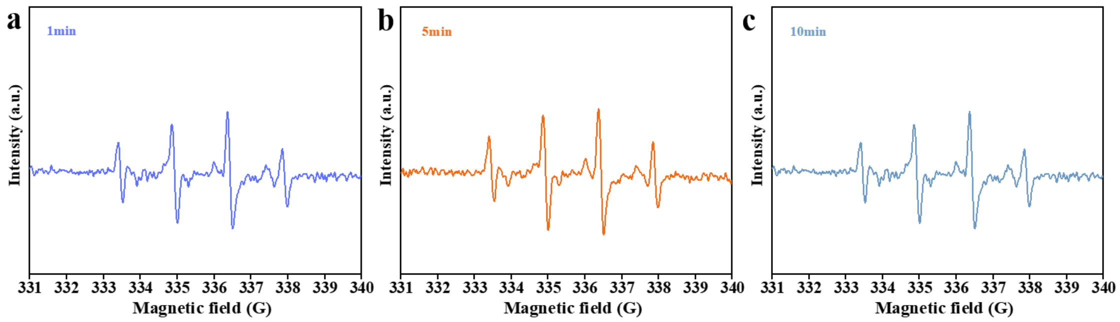


**Figure 13. ROS evaluation.** EPR spectra of $^{\bullet}OH$ and $SO_4^{\bullet-}$ radicals generated by the Co@VMT MEM/PMS system with different reaction time. Reaction conditions: [Ranitidine] = 10 ppm, [PMS] = 20 ppm, [DMPO] = 0.3 mM, pH = 4.0, T = 298 K, reaction time = 10 min).

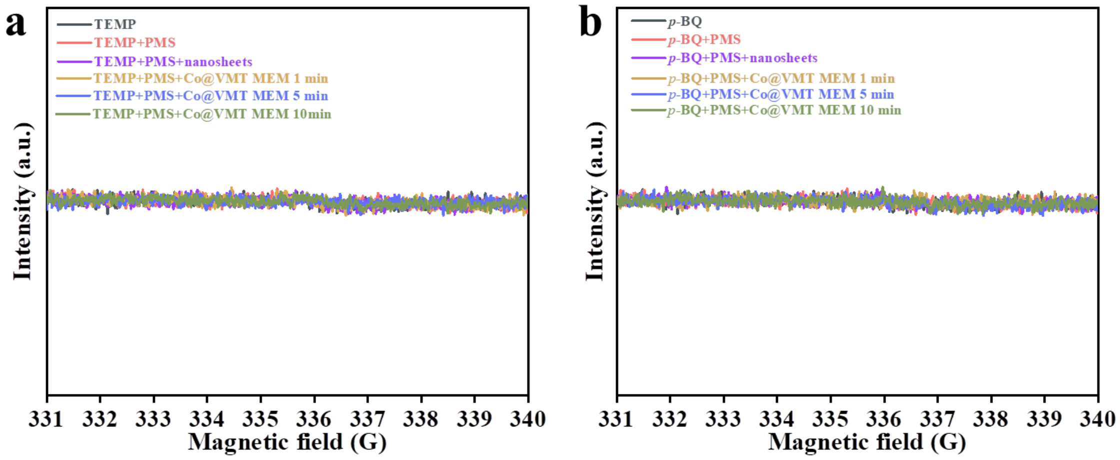


**Figure 14. ROS evaluation.** EPR spectra of $^1O_2$ (a) and $•O_2^-$ (b) radicals generated in both Co@VMT MEM/PMS and Co@VMT nanosheets/PMS systems. Reaction conditions: [Ranitidine] = 10 ppm, [PMS] = 20 ppm, [TEMP] = 50 mM, [p-BQ] = 5 mM pH = 4.0, T = 298 K).

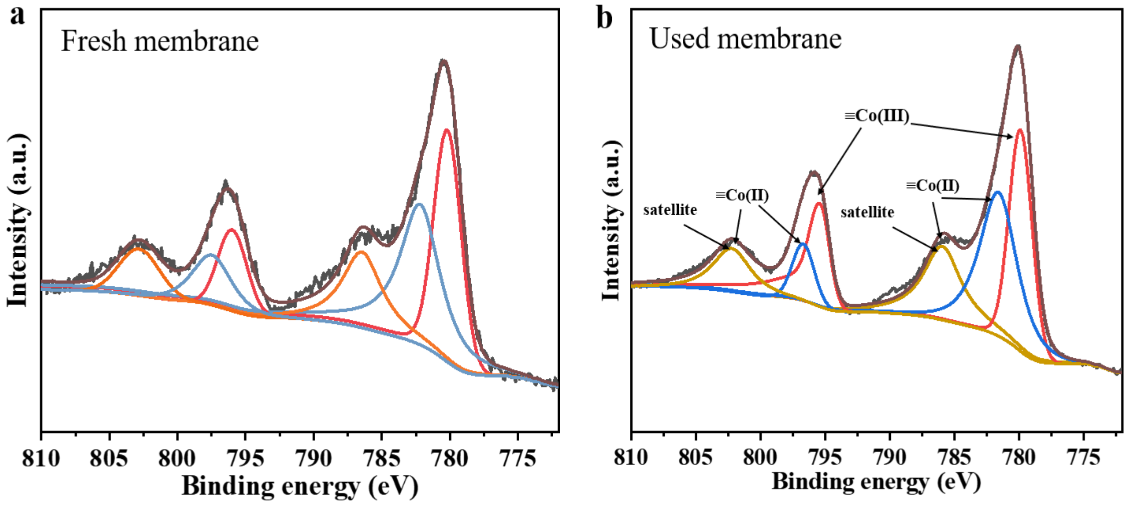


**Figure 15. Chemical stability evaluation.** Co 2p spectra of Co@VMT membrane before and after the 107 h stability experiment.

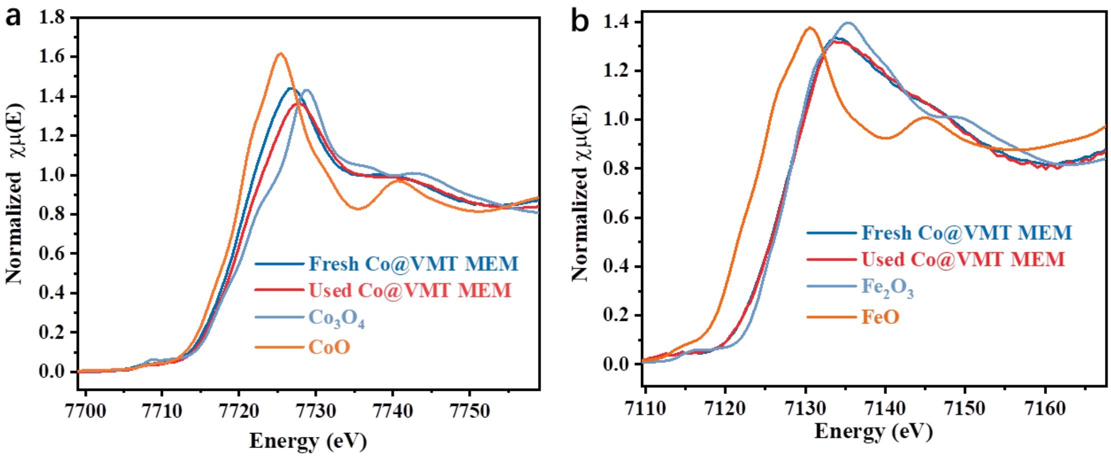


**Figure 16. Chemical stability evaluation.** Normalized XANES spectra at the Co K-edge (a) and Fe K-edge (b) of the fresh and used Co@VMT membrane.

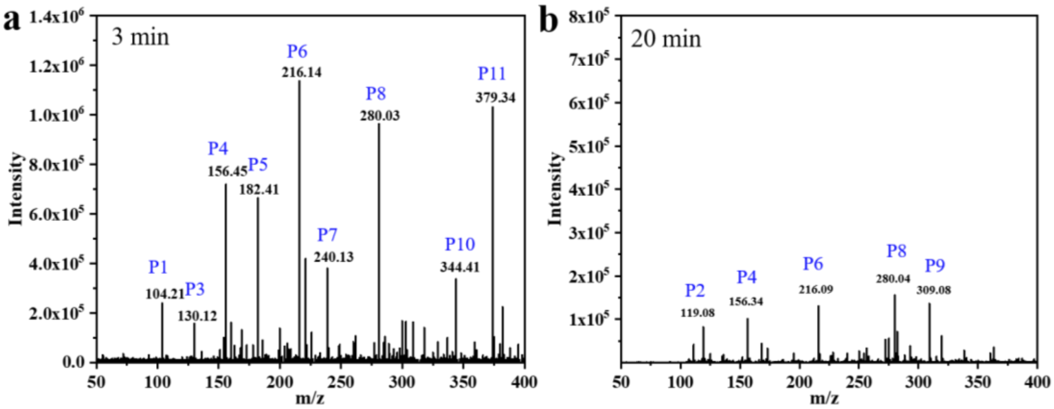


**Figure 17. Analysis of degradation intermediates**. Spectra of ranitidine degradation intermediates obtained in ESI (+)-MS mode by LC-MS for the Co@VMT membrane/PMS system at different reaction times of 3 min (a) and 20 min (b).

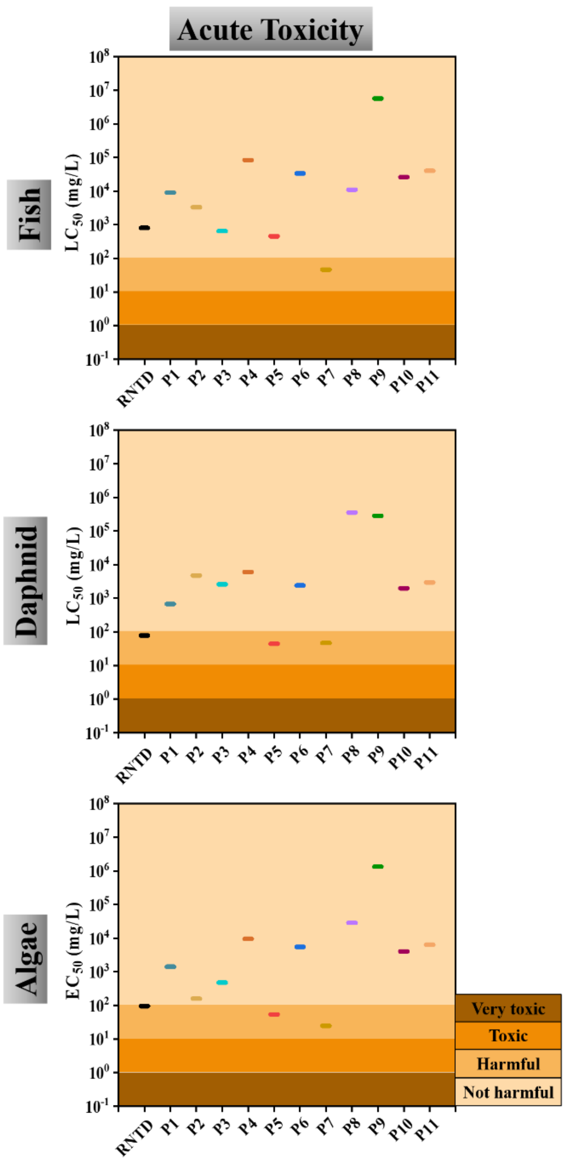


**Figure 18. Toxicity evaluation**. Toxicity estimation of ranitidine and its degradation intermediates using the ECOSAR program.

According to the Globally Harmonized System of Classification and Labeling of Chemicals (GHS), the predicted toxicity values of ranitidine and all intermediates can be divided into four categories: very toxic ($LC_{50}/EC_{50} < 1$ mg.L$^{-1}$), toxic (1 mg.L$^{-1}$ < $LC_{50}/EC_{50}$ < 10 mg.L$^{-1}$), harmful (10 mg.L$^{-1}$ < $LC_{50}/EC_{50}$ < 100 mg.L$^{-1}$), and not harmful ($LC_{50}/EC_{50}$ > 100 mg.L$^{-1}$)[3, 5].

## 3. Supplementary Tables

**Table 1.** EDX mapping results of Co@VMT membrane.

| Element | wt% | At% |
|---|---|---|
| O | 34.31 | 53.26 |
| Mg | 12.75 | 13.02 |
| Si | 23.8 | 21.05 |
| Fe | 16.71 | 7.43 |
| Co | 12.43 | 5.24 |

**Table 2.** Results of Co 2p XPS spectra of Co@VMT membrane.

| **Element** | **Bond type** | **E/eV** | **at/%** |
|---|---|---|---|
| Co 2p | ≡Co(II) | 802.82 | 7.33 |
| | | 797.48 | 8.28 |
| | | 786.25 | 14.63 |
| | | 782.18 | 32.07 |
| | ≡Co(III) | 795.95 | 9.08 |
| | | 780.18 | 28.61 |

**Table 3.** The best fitting parameters of coordination structure information derived from the Co K-edge FT-EXAFS fitting curve of Co@VMT membrane.

| Sample | Shell | N | R (Å) | $\Delta E_0$ | $\sigma^2$ ($\times 10^{-3}$ Å$^2$) | R factor |
|---|---|---|---|---|---|---|
| Co@VMT MEM | Co-O | 5.74 | 1.91 | -8.60 | 0.011 | 0.028 |
| | Co-O-Co | 0.68 | 2.87 | 6.00 | 0.008 | 0.028 |

**Table 4.** Surface area and pore volume analysis of VMT and Co@VMT membranes.

| Sample | $S_{BET}$ ($m^2.g^{-1}$) | $V_{total}$ ($cm^3.g^{-1}$) | t-plot | |
|---|---|---|---|---|
| | | | $S_{micro}$ ($m^2.g^{-1}$) | $S_{exter}$ ($m^2.g^{-1}$) |
| VMT membrane | 5.5329 | 0.015164 | 3.2077 | 2.3252 |
| VMT@Co membrane | 48.2768 | 0.108861 | 4.4434 | 43.8334 |

**Table 5.** Comparison of ranitidine removal efficiency over different technologies

| No. | Methods | Materials | Ranitidine concentration (mg.L$^{-1}$) | Ranitidine removal efficiency | Catalysts dosage | Feed solution pH | Reaction time | k (min$^{-1}$) | Ref |
|---|---|---|---|---|---|---|---|---|---|
| 1 | Photocatalysis | $MoS_2$/RGO | 10 | 74% | 1 g.L$^{-1}$ | 6.5 | 60 min | 0.0208 | 4 |
| 2 | Photocatalysis | $MoS_2$ | 10 | 33% | 1 g.L$^{-1}$ | 6.5 | 60 min | 0.00599 | 4 |
| 3 | Photocatalysis | RGO | 10 | 35% | 1 g.L$^{-1}$ | 6.5 | 60 min | 0.00644 | 4 |
| 4 | Photocatalysis | MXene-$Ti_3C_2$/$MoS_2$ | 10 | 88.40% | 1 g.L$^{-1}$ | 6.5 | 60 min | 0.03148 | 5 |
| 5 | Photocatalysis | MXene-$Ti_3C_2$ | 10 | 18.40% | 1 g.L$^{-1}$ | 6.5 | 60 min | 0.0032 | 5 |
| 6 | Photocatalysis | $TiO_2$ | 10 | 100% | 0.2 g.L$^{-1}$ | 6.5 | 45 min | 0.146 | 5 |
| 7 | Photo-Fenton | $TiO_2$+$Fe^{2+}$/$H_2O_2$ | 10 | 100% | 0.2 g.L$^{-1}$ | 6.6 | 22 min | 0.23 | 6 |
| 8 | Photocatalysis | $TiO_2$ nanofiber | 3 | 95% | -- | 6.5 | 120 min | 0.008 | 7 |
| 9 | Photocatalysis | Degussa P25 | 3 | 96% | -- | 6.5 | 120 min | 0.011 | 7 |
| 10 | UV photolysis | $NH_2Cl$ | 5 | 89.40% | 0.051-0.3 g.L$^{-1}$ | 6.0–8.0 | 5 min | 0.33 | 8 |
| 11 | Heterogeneous catalysis | $Co_3O_4$ NS | 5 | 47.20% | 0.02 g.L$^{-1}$ | 9.0 | 30 min | 0.021 | 9 |
| 13 | Heterogeneous catalysis | OM-$Co_3O_4$ | 10 | 99.20% | 0.25 g.L$^{-1}$ | 3.0 | 7 min | 0.719 | 10 |
| 14 | Heterogeneous catalysis | nZVIPs@$Ti_3C_2$ | 10 | 92.30% | 0.1 g.L$^{-1}$ | 3.0 | 6 min | 0.4311 | 11 |
| 15 | Heterogeneous catalysis | BN-$Co_3O_4$ | 10 | 99.60% | 0.03 g.L$^{-1}$ | 6.0 | 10 min | 0.682 | 12 |
| 16 | Nanoconfinement catalysis | Co@VMT MEM | 10 | 99.70–100% | 1.6 mg.cm$^{-2}$ | 4.0–9.0 | 5 min | 1.02 | This work |

**Table 6.** Operating conditions and the result of ICP-MS.

| | | | | |
|---|---|---|---|---|
| Operating conditions | RF Power | 1.5 kW | Plasma flow | 15.0 $L.min^{-1}$ |
| | Auxiliary flow | 1.0 $L.min^{-1}$ | Nebulizer flow | 0.10 $L.min^{-1}$ |
| | Sample untake delay | 30 s | Instr stabilization delay | 30 s |
| | Replicate read time | 5 s | Replicates | 3 times |
| **Result** | **Element** | **Co** | **Concentration** | **1.6 $\mu g.L^{-1}$** |

**Table 7.** Comparison of flux and rejection by the Co@VMT membrane/PMS system and the previously developed membranes in pollutant removal.

| No. | Membranes | Pollutant | Rejection (%) | Flux (L $m^{-2}$ $h^{-1}$) | Ref |
|---|---|---|---|---|---|
| 1 | MOF | Azithromycin | 97.6 | 15.4 | 13 |
| 2 | MOF | Diclofenac | 99.6 | 33 | 14 |
| 3 | MOF | Naproxen | 98.3 | 24.9 | 14 |
| 4 | Polymer | DB[b] | 99 | 20.3 | 15 |
| 5 | | MB[c] | 98 | 19.7 | 15 |
| 6 | MXene | RhB[d] | 99.8 | 86.5 | 16 |
| 7 | | MLB[e] | 99.5 | 88.8 | 16 |
| 8 | GO[a] | MB | 99.5 | 58.58 | 17 |
| 9 | GO | MB | 99.8 | 49.8 | 18 |
| 10 | $MoS_2$ | BPA[f] | 90 | 30.58 | 19 |
| 11 | Co@ VMT MEM | Ranitidine | 99.7 | 122.4 | This work |
| 12 | | MO[g] | 99.1 | 120.5 | |
| 13 | | MB | 98.9 | 119.25 | |
| 14 | | RhB | 99.7 | 119.5 | |
| 15 | | OXY[h] | 99.7 | 120.5 | |
| 16 | | CAR[i] | 99.3 | 121.25 | |
| 17 | | Phenol | 99 | 120.75 | |
| 18 | | BPA | 96.9 | 119.25 | |

[a]Graphene oxide; [b]Direct Black 38; [c]Methyl Blue; [d]Rhodamine B; [e]Methylene Blue; [f]Bisphenol A; [g]Methyl Orange; [h]Oxytetracycline; [i]Carbamazepine

**Table 8.** Specific water quality parameters for different real water matrices.

| Samples | Tap water | Lake water |
|---|---|---|
| pH | 7.14 | 6.89 |
| COD (mg.$L^{-1}$) | - | <15 |
| DOC (mg.$L^{-1}$) | - | 3.625 |
| $NO_3^-$ (mg.$L^{-1}$) | 1.35 | 0.25 |
| $Cl^-$ (mg.$L^{-1}$) | 12.8 | 4.47 |
| $CO_3^{2-}$ (mg.$L^{-1}$) | - | 13.36 |
| $PO_4^{3-}$ (mg.$L^{-1}$) | - | <0.01 |
| $NH_3$ (mg.$L^{-1}$) | - | 0.07 |
| Conductivity (μS.$cm^{-1}$) | 168.45 | 42.44 |

Note: "-" denotes not detected or the concentration of target parameter is below the detection limit of the device

**Table 9.** Results of XPS spectra of Co@VMT membranes before and after stability test.

| | | Before stability test | | | After stability test | | |
|---|---|---|---|---|---|---|---|
| **Element** | **Bond type** | **E/eV** | **at/%** | **Peak area** | **E/eV** | **at/%** | **Peak area** |
| Co 2p | ≡Co(Ⅱ) | 802.82 | 7.33 | 6550.86 | 802.2 | 8.62 | 32045.6 |
| | | 797.48 | 8.28 | 7435.86 | 796.68 | 5.86 | 21909.67 |
| | | 786.25 | 14.63 | 13300.33 | 785.91 | 15.04 | 56308.48 |
| | | 782.18 | 32.07 | 29280.72 | 781.96 | 29.21 | 110919.04 |
| | ≡Co(Ⅲ) | 795.95 | 9.08 | 8168.7 | 795.7 | 15.04 | 56308.48 |
| | | 780.18 | 28.61 | 26170.77 | 779.86 | 24.78 | 94259.59 |

**Table S10.** Degradation products of ranitidine in the Co@VMT/PMS system.

| **Compounds** | **m/z value** | **Structure** |
|---|---|---|
| Ranitidine | 315 | 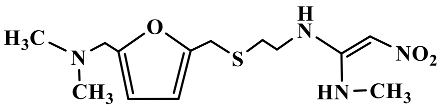 |
| P1 | 104 | 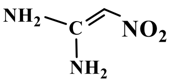 |
| P2 | 118 | 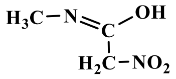 |
| P3 | 130 | 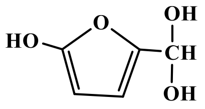 |
| P4 | 156 | 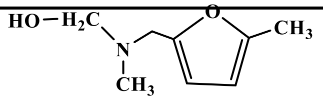 |
| P5 | 182 | 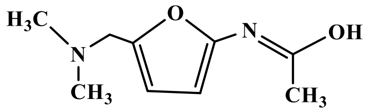 |
| P6 | 216 | 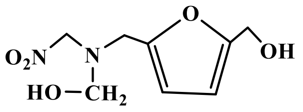 |
| P7 | 240 | 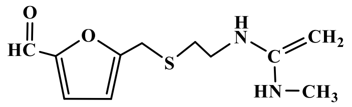 |
| P8 | 280 | 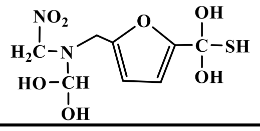 |
| P9 | 309 | 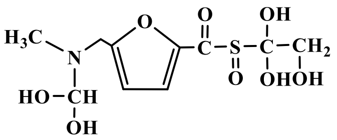 |
| P10 | 345 | 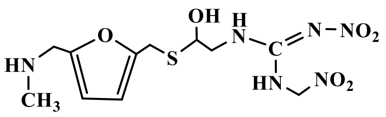 |
| P11 | 379 | 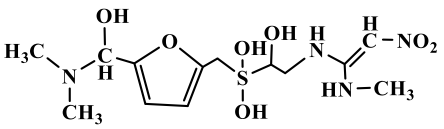 |

## Supplementary References


1. Meng C., Ding B., Zhang S., Cui L., Ostrikov K. K., Huang Z., Yang B., Kim J. H., Zhang Z., Angstrom-confined catalytic water purification within Co-$TiO_x$ laminar membrane nanochannels. *Nat Commun.* **13**, 4010 (2022).
2. Asif M. B., Zhang S., Qiu L., Zhang Z., Ultrahigh-permeance functionalized boron nitride membrane for nanoconfined heterogeneous catalysis. *Chem Catalysis* **2**, 550-562 (2022).
3. Persson L, Karlsson-Vinkhuyzen S, Lai A, Persson Å, Fick, S. The globally harmonized system of classification and labelling of chemicals—explaining the legal implementation gap. *Sustainability,* **9**, 2176 (2017).
4. Zou X, Zhang J, Zhao X, Zhang, Z. $MoS_2$/RGO composites for photocatalytic degradation of ranitidine and elimination of NDMA formation potential under visible light. *Chem. Eng. J.,* **383**, 123084 (2020).
5. Zou, X., Zhao, X., Zhang, J., Lv, W., Qiu, L., & Zhang, Z. Photocatalytic degradation of ranitidine and reduction of nitrosamine dimethylamine formation potential over MXene–$Ti_3C_2$/$MoS_2$ under visible light irradiation. *J. Hazard. Mater.,* **413**, 125424. (2021).
6. Radjenović J, Sirtori C, Petrović M, Barceló D, Malato, S. Characterization of intermediate products of solar photocatalytic degradation of ranitidine at pilot-scale. *Chemosphere,* **79**, 368-376 (2010).
7. Choi K J, Hong S W. Preparation of $TiO_2$ nanofibers immobilized on quartz substrate by electrospinning for photocatalytic degradation of ranitidine. *Res. Chem. Intermed.,* **38**, 1161-1169 (2012).
8. Wu Y, Zhu S, Wang J, Bu L, Deng J, Zhou, S. Role of reactive nitrogen species in ranitidine degradation in UV/chloramine process: Transformation pathways and NDMA formation. *Chem. Eng. J.,* **404**, 126557 (2021).
9. Ma Y, Lv X, Xiong D, Zhao X, Zhang Z. Catalytic degradation of ranitidine using novel magnetic Ti3C2-based MXene nanosheets modified with nanoscale zero-valent iron particles. *Appl. Catal. B.,* **284**, 119720 (2021).
10. Ma Y, Wang H, Lv X, Xiong D, Xie H, Zhang, Z. Three-dimensional ordered mesoporous $Co_3O_4$/peroxymonosulfate triggered nanoconfined heterogeneous catalysis for rapid removal

of ranitidine in aqueous solution. *Chem. Eng. J.,* **443,** 136495 (2022).

11. Ma Y, Xiong D, Lv X, Zhao X, Meng C, Xie H, Zhang, Z. Rapid and long-lasting acceleration of zero-valent iron nanoparticles@$Ti_3C_2$-based MXene/peroxymonosulfate oxidation with bi-active centers toward ranitidine removal. *J. Mater. Chem. A,* **9**, 19817-19833 (2021).
12. Ma Y, Ji B, Lv X, Xiong D, Zhao X, Xie H, Zhang, Z. Confined heterogeneous catalysis by boron nitride-$Co_3O_4$ nanosheet cluster for peroxymonosulfate oxidation toward ranitidine removal. *Chem. Eng. J.,* **435**, 135126. (2022).
13. Cheng X, Jiang X, Zhang Y, Lau C H, Xie Z, Ng D, Shao L. Building additional passageways in polyamide membranes with hydrostable metal organic frameworks to recycle and remove organic solutes from various solvents. *ACS Appl. Mater. Interfaces,* **9**, 38877-38886 (2017).
14. Paseta L, Antoran D, Coronas J, Tellez C. 110th anniversary: polyamide/metal–organic framework bilayered thin film composite membranes for the removal of pharmaceutical compounds from water. *Ind. Eng. Chem. Res.,* **58**, 4222-4230 (2019).
15. Zhang Y, Zhao K, Zhang Z, Xie H, Li Z, Lin Z, Wei J. Polypropylene non-woven supported calcium alginate hydrogel filtration membrane for efficient separation of dye/salt at low salt concentration. *Desalination,* **500**, 114845 (2021).
16. Long Q, Zhao S, Chen J, Zhang Z, Qi G, Liu Z Q. Self-assembly enabled nano-intercalation for stable high-performance MXene membranes. *J. Membr. Sci.,* **635**, 119464 (2021).
17. Chen L, Wang W, Fang Q, Zuo K, Hou G, Ai Q, Lou J. High performance hierarchically nanostructured graphene oxide/covalent organic framework hybrid membranes for stable organic solvent nanofiltration. *Appl. Mater. Today,* **20**, 100791 (2020).
18. Zhang W, Yin M, Zhao Q, Jin C, Wang N, Ji S, An Q. Graphene oxide membranes with stable porous structure for ultrafast water transport. *Nat. Nanotechnol.,* **16**, 337-343 (2021).
19. Chen Y, Zhang G, Liu H, Qu J. Confining free radicals in close vicinity to contaminants enables ultrafast fenton-like processes in the interspacing of $MoS_2$ membranes. *Angew. Chem. Int. Ed.,* **58**, 8134-8138 (2019).
