# Supplementary material for: Overcoming the permeability-selectivity challenge in water purification using two-dimensional cobalt-functionalized vermiculite membrane
Source: Nat Commun. 2024 Jan 9;15:391. doi: 10.1038/s41467-024-44699-0 (PMC10776859; doi:10.1038/s41467-024-44699-0)
Supplement: Supplementary file 3 — Description of Additional Supplementary Files [file 41467_2024_44699_MOESM3_ESM.pdf]

Description of Additional Supplementary Files

File Name: Supplementary Movie 1
Description: MD simulations of mass transfer within VMT membrane.

File Name: Supplementary Movie 2
Description: MD simulations of mass transfer within Co@VMT membrane.
